# Antibiotic Resistance and Molecular Biological Characteristics of Non-13-Valent-Pneumococcal Conjugate Vaccine Serogroup 15 *Streptococcus pneumoniae* Isolated From Children in China

**DOI:** 10.3389/fmicb.2021.778985

**Published:** 2022-01-05

**Authors:** Wei Shi, Qianqian Du, Lin Yuan, Wei Gao, Qing Wang, Kaihu Yao

**Affiliations:** Beijing Key Laboratory of Pediatric Respiratory Infection Diseases, Key Laboratory of Major Diseases in Children, Ministry of Education, National Key Discipline of Pediatrics (Capital Medical University), National Clinical Research Center for Respiratory Diseases, National Center for Children’s Health, Beijing Pediatric Research Institute, Beijing Children’s Hospital, Capital Medical University, Beijing, China

**Keywords:** *Streptococcus pneumoniae*, serotype 15, PCV13, vaccine, antibiotic resistance, children

## Abstract

**Background:** The isolation rate of serogroup 15 *Streptococcus pneumoniae* has been increasing since developing countries began administering the 13-valent pneumococcal conjugate vaccine.

**Methods:** We detected the antibiotic resistance and molecular characteristics of 126 serogroup 15 *S. pneumoniae* strains isolated from children in China. Serotypes were determined via the Quellung reaction. Antibiotic resistance was tested using the *E*-test or disc diffusion method. Sequence types were assigned via multilocus sequence typing. Data were analyzed using WHONET 5.6 software.

**Results:** The frequencies of *S. pneumoniae* serotypes 15A, 15B, 15C, and 15F were 29.37, 40.48, 28.57, and 1.59%, respectively. Continuous-monitoring data from Beijing showed that the annual isolation rates of serogroup 15 *S. pneumoniae* were 7.64, 7.17, 2.58, 4.35, 3.85, 7.41, and 10.53%, respectively, from 2013 to 2019. All 126 serogroup 15 strains were susceptible to vancomycin and ceftriaxone. The non-susceptibility rate to penicillin was 78.57%. All strains were resistant to erythromycin with high minimum inhibitory concentrations (MICs). The multidrug resistance rate was 78.57%. The most common clonal complexes were CC3397, CC6011, CC10088, CC9785, and ST8589.

**Conclusion:** Serogroup 15 *S. pneumoniae* is common among children in China, and these strains should be continuously monitored.

## Introduction

*Streptococcus pneumoniae* (*S. pneumoniae*) is among the most important pathogenic bacteria in children. More than 100 serotypes within 46 serogroups have been discovered according to the biochemical structure of the capsular polysaccharide (Ganaie et al., 2020). As of 12 October 2020, the 13-valent pneumococcal conjugate vaccine (PCV13), which protects against 13 serotypes (1, 3, 4, 5, 6A, 6B, 7F, 9V, 14, 18C, 19A, 19F, and 23F) has been incorporated into national immunization programs in 160 countries (World Health Organization, 2020). Research on the efficacy of PCV13 showed that PCV13 can reduce the incidence of pneumococcal disease and cause changes in the serotype distributions of pathogenic *S. pneumoniae* strains (Janoir et al., 2016; Schroeder et al., 2017; Ubukata et al., 2018; Kim et al., 2020). Many regions worldwide have reported increased isolation rates of serogroup 15 *S. pneumoniae* strains after PCV13 use (Linden et al., 2015; Liyanapathirana et al., 2015; Sheppard et al., 2016; Lo et al., 2019; Nakano et al., 2020).

PCV13 was approved in China in November 2016 and was available in the country in May 2017. Because PCV13 is expensive and has not been included in China’s national immunization program, the PCV13 vaccination rate among Chinese children is low. Another 23-valent pneumococcal polysaccharide vaccine, PPV23, which protects against serotypes 1, 2, 3, 4, 5, 6B, 7F, 8, 9N, 9V, 10A, 11A, 12F, 14, 15B, 17F, 18C, 19A, 19F, 20, 22F, 23F, and 33F, is available in China for children aged > 2 years. The vaccination rates for both vaccines are low. Recipients are concentrated in cities and are children whose parents can afford to pay for the vaccines. Reports on the vaccination data are scarce, and the vaccine coverage is unclear.

We sought to determine the prevalence of serogroup 15 *S. pneumoniae* among Chinese children as well as the characteristics of these strains. Here, we report the serotype distribution, antibiotic-resistance patterns and molecular biological characteristics of 126 serogroup 15 *S. pneumoniae* strains collected from children in China.

## Materials and Methods

### Bacterial Strains

We collected 126 unduplicated strains of serogroup 15 *S. pneumoniae* from Beijing (Beijing Children’s Hospital affiliated to Capital Medical University; hereinafter referred to as Beijing Children’s Hospital), Zhongjiang (People’s Hospital of Zhongjiang County), Youyang (People’s Hospital of Chongqing Youyang County), Wulumuqi (Wulumuqi Children’s Hospital) and Shenzhen (Shenzhen Children’s Hospital) from 2013 to 2019. Table 1 lists the detailed clinical information on these children, including age, sex, culture time and specimen type isolated.

**TABLE 1:** Clinical information on the strains included in this study.

	Beijing (*n* = 63)	Youyang (*n* = 27)	Zhongjiang (*n* = 17)	Wulumuqi (*n* = 13)	Shenzhen (*n* = 6)	Total (*n* = 126)
**Age (year)**						
≤ 1	32	22	11	7	1	73 (57.94%)
∼2	7	2	1	0	0	10 (7.94%)
∼3	9	1	1	2	3	16 (12.70%)
∼4	3	2	2	1	2	10 (7.94%)
∼5	4	0	2	2	0	8 (6.35%)
>5	8	0	0	1	0	9 (7.14%)
**Gender**						
Male	36	17	12	6	4	75 (59.52%)
Female	27	10	5	7	2	51 (40.48%)
**Collecting year**						
2013	11	–	–	–	–	11 (8.73%)
2014	16	2	1	–	–	19 (15.08%)
2015	4	25	16	1	–	46 (36.51%)
2016	6	–	–	2	–	8 (6.35%)
2017	4	–	–	–	–	4 (3.17%)
2018	14	–	–	10	6	30 (23.81%)
2019	8	–	–	–	–	8 (6.35%)
**Specimen types**						
Nasopharyngeal swabs	12	27	17	3	6	65 (51.59%)
Sputum	29	–	–	10	–	39 (30.95%)
Bronchoalveolar lavage fluid	19	–	–	–	–	19 (15.08%)
Venous blood	2	–	–	–	–	2 (1.59%)
Cerebrospinal fluid	1	–	–	–	–	1 (0.79%)

Of the 126 strains isolated, 63 were collected via continuous surveillance in Beijing Children’s Hospital. Beijing Children’s Hospital is a national children’s medical center, with more than 3 million outpatients and 70,000 inpatients annually. Since 2013, Beijing Children’s Hospital has been monitoring the serotype and antibiotic susceptibility characteristics of *S. pneumoniae* strains isolated from children in the hospital. In this study, the isolation rate of serogroup 15 *S. pneumoniae* strains from Beijing Children’s Hospital was used to reflect the isolation of serogroup 15 *S. pneumoniae* in children in China.

A parent and/or legal guardian of each participant signed a written informed consent document before enrollment and before the study procedures were performed. The Ethics Committees of Beijing Children’s Hospital affiliated to Capital Medical University, People’s Hospital of Zhongjiang County, People’s Hospital of Chongqing Youyang County, Wulumuqi Children’s Hospital, and Shenzhen Children’s Hospital approved the study. No ethical problems were encountered.

### Serotyping

Serotypes were determined using the Quellung reaction with Pneumotest kits (Statens Serum Institute, Copenhagen, Denmark). Serotyping was interpreted based on capsular swelling under phase-contrast microscopy with an oil-immersion lens (magnification, 100×), as described previously (Sørensen, 1993).

### Antimicrobial Susceptibility Testing

The minimum inhibitory concentrations (MICs) of penicillin, amoxicillin, ceftriaxone, imipenem, vancomycin and erythromycin for each strain were determined using *E*-test strips (AB Biodisk, Solna, Sweden). Disc diffusion tests (Oxoid Ltd., Basingstoke, United Kingdom) were performed to ascertain antimicrobial susceptibilities to tetracycline, sulfamethoxazole-trimethoprim, and chloramphenicol. The results were interpreted in accordance with the Clinical and Laboratory Standards Institute 2019 guidelines, and oral breakpoints (susceptible, ≤ 0.06 mg/L; intermediate, 0.12–1.0 mg/L; resistant, ≥ 2.0 mg/L) were used for penicillin (Clinical Laboratory Standards Institute [CLSI], 2019). *S. pneumoniae* American Type Culture Collection 49619 was used as the quality-control strain and was included in each test set to ensure accuracy of the results. Multidrug-resistant *S. pneumoniae* (MDRSP) was defined as *S. pneumoniae* isolates that were resistant to three or more kinds of antibiotics tested in this study.

### Multilocus Sequence Typing

Strains were characterized using multilocus sequence typing (MLST). Chromosomal DNA was extracted from overnight cultures of *S. pneumoniae* strains grown on 5% trypticase soy agar (Oxoid Ltd.) using the SiMax™ Genomic DNA Extraction Kit (SBS Genetech Co., Ltd., Beijing, China) per the manufacturer’s instructions. Seven housekeeping genes (*aroE*, *gdh*, *gki*, *recP*, *spi*, *xpt*, and *ddl*) were amplified via polymerase chain reaction from the chromosomal DNA as described previously (Enrigh and Spratt, 1998). The products were sent to BGI Company (Beijing, China) for sequencing of both strands. The resulting sequences were compared with those of all known alleles at each locus, as well as with the sequence types (STs) in the pneumococcal MLST database.^1^ New alleles and allelic profiles were submitted to the MLST database for name assignment. goeBURST v1.2.1^2^ was used to investigate relationships among the strains and assign strains to a clonal complex (CC) based on the stringent group definition of six of the seven shared alleles.

### Statistical Analysis

Antimicrobial susceptibility and MLST data were analyzed using WHONET 5.6 as recommended by the World Health Organization.

## Results

### Serotype Distribution and Antimicrobial Susceptibility

Among the 126 strains, the proportions of serotypes 15A, 15B, 15C, and 15F were 29.37% (37/126), 40.48% (51/126), 28.57% (36/126), and 1.59% (2/126), respectively. Table 2 shows the antimicrobial susceptibility testing results, including MIC values, for the serotypes. All strains were susceptible to vancomycin and ceftriaxone; none were susceptible to amoxycillin. The non-susceptibility rate to penicillin was 78.57%. All 126 strains were resistant to erythromycin, with high MIC values of 24 mg/L for one strain and > 256 mg/L for all other strains. The resistance rate to tetracycline reached 92.86%.

**TABLE 2:** Antimicrobial susceptibility pattern of the 126 serogroup 15 *Streptococcus pneumoniae* strains.

Antimicrobial	Parameter	Isolates n (%)				
		Total (*n* = 126)	15A (*n* = 37)	15B (*n* = 51)	15C (*n* = 36)	15F (*n* = 2)
Penicillin	S	27 (21.43)	17 (45.95)	4 (7.84)	5 (13.89)	1 (50.00)
	I	55 (43.65)	18 (48.65)	24 (47.06)	13 (36.11)	0
	R	44 (34.92)	2 (5.41)	23 (45.10)	18 (50.00)	1 (50.00)
	MIC50	1	0.125	1	1	–
	MIC90	2	1	2	2	–
	MIC range	<0.016–4	<0.016–2	<0.016–4	<0.016–4	0.016, 2
Amoxicillin	S	116 (92.06)	37 (100.00)	45 (88.24)	32 (88.89)	2 (100.00)
	I	10 (7.94)	0	6 (11.76)	4 (11.11)	0
	R	0	0	0	0	0
	MIC50	1	0.25	1	2	–
	MIC90	4	1	4	4	–
	MIC range	<0.016–4	<0.016–2	<0.016–4	<0.016–4	0.016, 2
Ceftriaxone	MIC50	0.5	0.125	1	1	–
	MIC90	1	1	1	1	–
	MIC range	0.016–1	0.016–1	0.016–1	0.016–1	0.016, 1
Imipenem	S	44 (34.92)	25 (67.57)	10 (19.61)	8 (22.22)	1 (50.00)
	I	71 (56.35)	12 (32.43)	35 (68.63)	23 (63.89)	1 (50.00)
	R	11 (8.73)	0	6 (11.76)	5 (13.89)	0
	MIC50	<0.016	0.064	0.25	0.25	–
	MIC90	0.5	0.5	1	1	–
	MIC range	<0.016–1	<0.016–0.5	<0.016–1	<0.016–1	0.016, 0.5
Vancomycin	MIC50	0.5	0.5	0.5	0.5	1
	MIC90	1	1	1	1	1
	MIC range	0.5–1	0.5–1	0.5–1	0.5–1	1
Erythromycin	MIC50	> 256	> 256	> 256	> 256	> 256
	MIC90	> 256	> 256	> 256	> 256	> 256
	MIC range	24– > 256	24– > 256	> 256	>256	> 256
Tetracycline	S	6 (4.76)	5 (13.51)	0	0	1 (50.00)
	I	3 (2.38)	1 (2.70)	1 (1.96)	1 (2.78)	0
	R	117 (92.86)	31 (83.78)	50 (98.04)	35 (97.22)	1 (50.00)
Trimethoprim/sulfamethoxazole	S	33 (26.19)	32 (86.49)	0	0	1 (50.00)
	I	4 (3.17)	2 (5.41)	1 (1.96)	1 (2.78)	0
	R	89 (70.63)	3 (8.11)	50 (98.04)	35 (97.22)	1 (50.00)
Chloramphenicol	S	115 (91.27)	35 (94.59)	47 (92.16)	31 (86.11)	2 (100.00)
	I	0	0	0	0	0
	R	11 (8.73)	2 (5.41)	4 (7.84)	5 (13.89)	0

*MIC, minimum inhibitory concentrations.*

**FIGURE 1:**
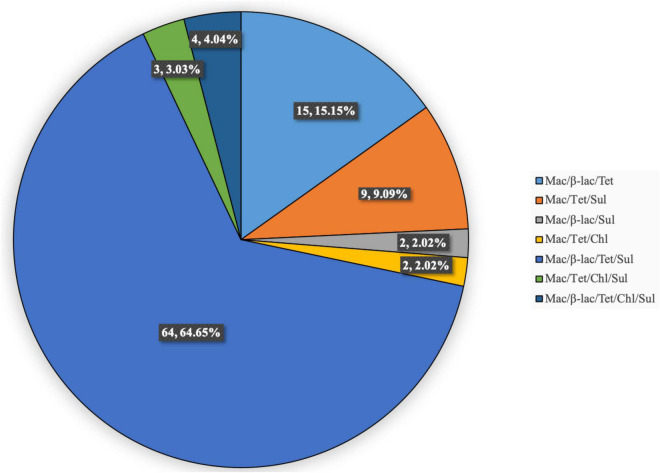
Distribution of antibiotic-resistance patterns of MDRSP strains. Mac, macrolides; β-lac, β-lactams; Tet, tetracyclines; Sul, sulfonamides; Chl, chloramphenicols.

The multidrug-resistance rate was 78.57% (99/126). Figure 1 shows the antibiotic-resistance pattern distributions of the MDRSP strains. The most common multidrug-resistance pattern was macrolides-β-lactams-tetracyclines, accounting for 64.65% (64/99) of the MDRSP.

### Multilocus Sequence Typing

**FIGURE 2:**
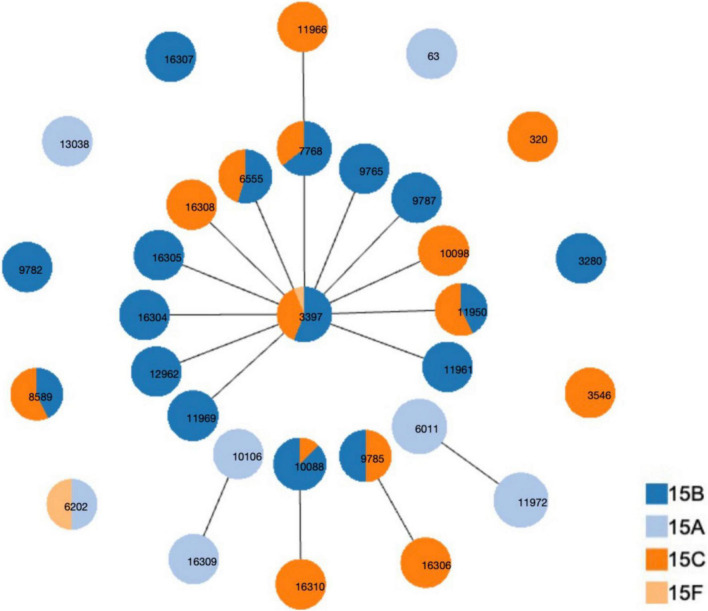
Distribution of sequence types (STs) amongst the clonal complexes of serogroup 15 *Streptococcus pneumoniae* strains. goeBURST analysis was performed on the multilocus sequence typing data generated from all 126 strains analyzed. STs linked by a line belong to the same cluster.

Thirty-one STs were identified among the 126 strains; the most common were ST3397 (*n* = 16, 12.70%), ST6011 (*n* = 16, 12.70%), ST11972 (*n* = 15, 11.90%), ST7768 (*n* = 14, 11.11%), ST6555 (*n* = 11, 8.73%), ST10088 (*n* = 8, 6.35%), ST8589 (*n* = 7, 5.56%), ST11950 (*n* = 7, 5.56%), and ST9785 (*n* = 6, 4.76%). The 31 STs were assigned to five CCs and nine singletons using goeBURST analysis (Figure 2). The most predominant CCs were CC3397 (*n* = 60, 47.62%) and CC6011 (*n* = 31, 24.60%), constituting 72.22% of all strains.

Table 3 shows the CCs/ST distribution by serotype. Each serotype had a predominant CC that included most of the isolates. CC6011 was the main CC for the 15A strains (83.78%, 31/37). CC3397 was the main CC for 15B and 15C, accounting for 68.63% (35/51) and 66.67% (24/36) of these strains, respectively.

**TABLE 3:** CC/ST distribution of serogroup 15 *S. pneumoniae* by serotype.

Serotypes	No.	CC3397	CC6011	CC10088	CC9785	ST8589	Others
15A	37	0	31 (83.78%)	0	0	0	6 (16.22%)
15B	51	35 (68.63%)	0	7 (13.73%)	3 (5.88%)	3 (5.88%)	3 (5.88%)
15C	36	24 (66.67%)	0	2 (5.56%)	4 (11.11%)	4 (11.11%)	2 (5.56%)
15F	2	1 (50.00%)	0	0	0	0	1 (50.00%)
Total	126	60	31	9	7	7	12

*%, number of serotypes divided by number of CCs/STs.*

### Relationship Between Antimicrobial Susceptibility and Clonal Complexes

Table 4 summarizes the antibiotic-resistance profiles according to CCs. Different CCs showed different antimicrobial-resistance characteristics. No CC3397 strains were susceptible to penicillin or tetracycline, and all were 100% resistant to sulfamethoxazole-trimethoprim and 100% sensitive to chloramphenicol. All CC6011 and CC10088 strains were susceptible to amoxicillin and resistant to imipenem. All CC10088 strains were intermediate to penicillin, resistant to tetracycline and susceptible to chloramphenicol. All CC9785 strains were resistant to penicillin. All ST8589 strains were susceptible to penicillin, amoxicillin, and imipenem and resistant to tetracycline, sulfamethoxazole-trimethoprim, and chloramphenicol.

**TABLE 4:** Antimicrobial susceptibility of the 126 strains by clonal complex.

Antimicrobial	Parameter	Isolates n (%)						
		Total (*n* = 126)	CC3397 (*n* = 60)	CC6011 (*n* = 31)	CC10088 (*n* = 9)	CC9785 (*n* = 7)	ST8589 (*n* = 7)	Others (*n* = 12)
Penicillin	S	27 (21.43)	0	15 (48.39)	0	0	7 (100.00)	5 (41.67)
	I	55 (43.65)	27 (45.00)	15 (48.39)	9 (100.00)	0	0	4 (33.33)
	R	44 (34.92)	33 (55.00)	1 (3.33)	0	7 (100.00)	0	3 (25.00)
	MIC50	1	2	0.125	1	2	<0.016–0.064	0.125
	MIC90	2	2	1	1	4	0.032	2
	MIC Range	<0.016–4	0.094–2	<0.016–2	0.5–1	2–4	0.064	<0.016–2
Amoxycillin	S	116 (92.06)	51 (85.00)	31 (100.00)	9 (100.00)	7 (100.00)	7 (100.00)	11 (91.67)
	I	10 (7.94)	9 (15.00)	0	0	0	0	1 (8.33)
	R	0	0	0	0	0	0	0
	MIC50	1	2	0.5	0.125	2	<0.016	0.023
	MIC90	4	4	1	0.25	2	<0.016	2
	MIC Range	<0.016–4	<0.016–4	<0.016–1	0.125–0.25	1–2	<0.016	<0.016–4
Ceftriaxone	MIC50	0.5	1	0.25	0.5	1	0.032	0.064
	MIC90	1	1	1	0.5	1	0.032	1
	MIC Range	0.016–1	0.5–1	0.016–1	0.25–0.5	0.5–1	0.016–0.032	0.016–1
Imipenem	S	44 (34.92)	2 (3.33)	20 (64.52)	6 (66.67)	1 (14.29)	7 (100.00)	8 (66.67)
	I	71 (56.35)	50 (83.33)	11 (35.48)	3 (33.33)	3 (42.86)	0	4 (33.33)
	R	11 (8.73)	8 (13.33)	0	0	3 (42.86)	0	0
	MIC50	<0.016	0.5	0.125	0.125	1	<0.016	0.064
	MIC90	0.5	1	0.5	0.25	1	<0.016	0.25
	MIC Range	<0.016–1	<0.016–1	<0.016–0.5	0.064–0.25	0.125–1	<0.016	<0.016–0.5
Vancomycin	MIC50	0.5	1	0.5	0.5	0.5	1	0.5
	MIC90	1	1	1	1	1	1	1
	MIC Range	0.5–1	0.5–1	0.5–1	0.5–1	0.5–1	0.5–1	0.5–1
Erythromycin	MIC50	> 256	> 256	> 256	> 256	> 256	> 256	> 256
	MIC90	> 256	> 256	> 256	> 256	> 256	> 256	> 256
	MIC Range	24– > 256	> 256	>256	> 256	>256	> 256	24– > 256
Tetracycline	S	6 (4.76)	0	3 (9.68)	0	0	0	3 (25.00)
	I	3 (2.38)	1 (1.67)	1 (3.23)	0	1 (14.29)	0	0
	R	117 (92.86)	59 (98.33)	27 (87.10)	9 (100.00)	6 (85.71)	7 (100.00)	9 (75.00)
Trimethoprim/sulfamethoxazole	S	33 (26.19)	0	29 (93.55)	0	0	0	4 (33.33)
	I	4 (3.17)	0	1 (3.23)	1 (11.11)	0	0	2 (16.67)
	R	89 (70.63)	60 (100.00)	1 (3.23)	8 (88.89)	7 (100.00)	7 (100.00)	6 (50.00)
Chloramphenicol	S	115 (91.27)	60 (100.00)	30 (96.77)	9 (100.00)	7 (100.00)	0	9 (75.00)
	I	0	0	0	0	0	0	0
	R	11 (8.73)	0	1 (3.23)	0	0	7 (100.00)	3 (25.00)

*MIC, minimum inhibitory concentrations.*

### Fluctuation of Serotypes by Years

**FIGURE 3:**
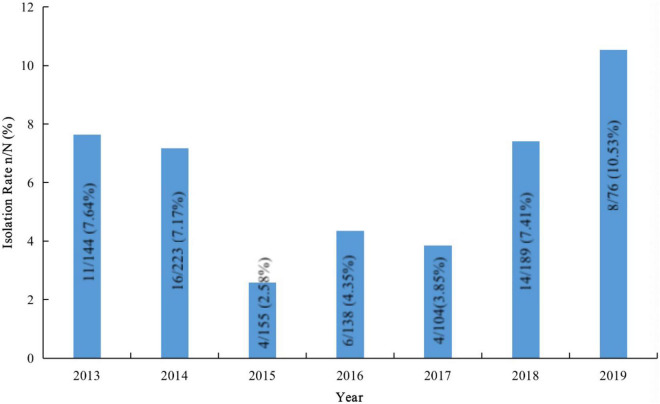
Isolation rates of serogroup 15 *Streptococcus pneumoniae* strains in samples from Beijing Children’s Hospital.

We isolated 1029 *S. pneumoniae* strains from Beijing Children’s Hospital during the study period, and 63 were determined to be serogroup 15. The proportion of serogroup 15 *S. pneumoniae* strains was 6.12%. Figure 3 shows the number of *S. pneumoniae* strains collected yearly and the serogroup 15 isolation rates.

In 2014 and 2015, the proportions of serogroup 15 *S. pneumoniae* were 6.67% (2/30) and 12.20% (25/205), respectively, in Youyang and 6.25% (1/16) and 9.09% (16/176), respectively, in Zhongjiang. In 2015, 2016, and 2018, the proportions of serogroup 15 were 6.25% (1/16), 12.50% (2/16), and 3.88% (10/258), respectively, in Wulumuqi. Six of 66 (9.09%) strains were isolated in Shenzhen in 2018.

## Discussion

Since the introduction of PCV13, many countries and regions that included PCV13 in their national immunization programs have reported increased infections from serogroup 15 *S. pneumoniae*, which is not covered by the PCV13 vaccine (Linden et al., 2015; Sheppard et al., 2016; Nakano et al., 2020). Serogroup 15 *S. pneumoniae* strains have caused outbreaks (Katherine et al., 2012) and deaths (Arushothy et al., 2020) among children. China has not yet included PCV13 in the national immunization program, and the PCV13 vaccination rate among Chinese children is low. Additionally, the epidemiological monitoring data for *S. pneumoniae* in Chinese children are inexact; thus, the prevalence of serogroup 15 *S. pneumoniae* and the characteristics of these strains are unclear. Here, we reported the serotype distribution, antibiotic-resistance patterns, and ST characteristics of 126 serogroup 15 *S. pneumoniae* strains isolated from children in China. Half of these strains came from the continuous monitoring of Beijing Children’s Hospital, which is one of the few continuous-monitoring data programs in mainland China and can partially reflect the prevalence of *S. pneumoniae* in Chinese children. During the study period, the overall isolation rate of serogroup 15 *S. pneumoniae* in the child population of Beijing Children’s Hospital was 6.12%. After PCV13 was launched in China in May 2017, the isolation rates of serogroup 15 *S. pneumoniae* in 2018 and 2019 were 7.41 and 10.53%, respectively, showing an increasing trend.

The Pneumococcal Molecular Epidemiology Network (PMEN)^3^ was established in 1997 for global surveillance of antibiotic-resistant *S. pneumoniae* and to standardize the nomenclature and classification of resistant clones. One 15A serotype was included among the 43 drug-resistant clones spread worldwide and published by the PMEN: Sweden^15A^-25 (ST63). Over the past few years, several studies have reported increasing numbers of serotype 15A-ST63 strains (Ho et al., 2015; Sheppard et al., 2016; Nakano et al., 2018, 2019, 2020). In this study, ST63 accounted for 5.41% (2/37) of the serotype 15A strains, which was far lower than the 88.24% reported in Hong Kong (Ho et al., 2015) and the 98.28% reported in Japan (Nakano et al., 2020). Some studies reported that ST63 showed high multidrug-resistance rates (Sheppard et al., 2016; Nakano et al., 2018). Both ST63 strains in this study were resistant to erythromycin; one of these was also resistant to tetracycline, and the other was non-susceptible to imipenem, but both strains were sensitive to other antibiotics. The significant differences in isolation rates and antibiotic susceptibility characteristics of ST63 between our study and the other studies may be due to regional differences.

In the present study, most of the serotype 15A strains belonged to CC6011, which was composed of ST6011 and ST11972. ST6011 was popular in both serotype 3 (Ho et al., 2019) and serogroup 15 strains. There were 28 ST6011 strains recorded in the MLST database,^4^ among which 22 were serotype 3, 5 were serotype 15A, and 1 strain was serotype 15B/15C. Both serotype 3 and serogroup 15 are non-PCV13 vaccine serotypes, and have an increasing trend in recent years (Linden et al., 2015; Liyanapathirana et al., 2015; Sheppard et al., 2016; LeBlanc et al., 2019; Lo et al., 2019; Goettler et al., 2020; Nakano et al., 2020). Therefore, close attention should be paid to ST6011 prevalent in these two serotypes.

We found that serogroup 15 *S. pneumoniae* strains showed good sensitivity to common antibiotics. However, the most common CC, CC3397, was 100% non-susceptible to penicillin. We wondered whether the prevalence of CC3397 in serogroup 15 *S. pneumoniae* results from antibiotic pressure. Many studies have reported clonal shift phenomena in other serotypes, as CC271 replaced CC983 among serotype 19F strains (Li et al., 2013), ST81 replaced ST342 amongst serotype 23F pneumococcal isolates (Ma et al., 2013), CC320 replaced CC230 in 19A strains (Beall et al., 2011; Golden et al., 2015; Mayanskiy et al., 2017), and CC876 replaced CC875 in serotype 14 strains (He et al., 2015). These examples of clonal shift phenomena in the same serotype may be caused by antibiotic pressure, as they followed the principle that CCs/STs expressing high antibiotic resistance will replace CCs/STs with lower antibiotic resistance. Presently, the epidemiological characteristics of serogroup 15 *S. pneumoniae* are not being continuously monitored to access the changes in CC/ST prevalence in China. Whether CC3397 is the most drug-resistant of the serogroup 15 *S. pneumoniae* clones or whether CC3397 will be replaced by more drug-resistant clones in the future is unclear. Therefore, clinicians must strengthen surveillance of the antibiotic resistance and other epidemiological characteristics of serogroup 15 *S. pneumoniae*.

## Conclusion

Serogroup 15 *S. pneumoniae* is common among children in China. Because CC3397, the main CC of serogroup 15 *S. pneumoniae*, showed high non-susceptibility to penicillin, long-term monitoring of antibiotic susceptibility and other epidemiological characteristics of serogroup 15 *S. pneumoniae* is essential.

## Data Availability Statement

The original contributions presented in the study are included in the article/supplementary material, further inquiries can be directed to the corresponding author/s.

## Author Contributions

WS, QD, LY, and WG conducted the experiments. WS and QW were responsible for the laboratory analysis. WS and KY designed the study, collected the data, analyzed the data, interpreted the results, and drafted the manuscript. All authors reviewed and revised the manuscript and approved the final version.

## Conflict of Interest

The authors declare that the research was conducted in the absence of any commercial or financial relationships that could be construed as a potential conflict of interest.

## Publisher’s Note

All claims expressed in this article are solely those of the authors and do not necessarily represent those of their affiliated organizations, or those of the publisher, the editors and the reviewers. Any product that may be evaluated in this article, or claim that may be made by its manufacturer, is not guaranteed or endorsed by the publisher.

## Footnotes

1
https://pubmlst.org/spneumoniae/

2
http://www.phyloviz.net/goeburst/

3
https://www.pneumogen.net/pmen

4
https://pubmlst.org
